# Dis/re-orienting design through norm-critical gender lenses: an educational case in Turkey

**DOI:** 10.3389/fsoc.2024.1341091

**Published:** 2024-03-28

**Authors:** Erman Örsan Yetiş, Yekta Bakırlıoğlu

**Affiliations:** ^1^Department of Politics and International Relations, The University of Sheffield, Sheffield, United Kingdom; ^2^Imagination Lancaster, Lancaster University, Lancaster, United Kingdom; ^3^Department of Industrial Design, Middle East Technical University, Ankara, Türkiye

**Keywords:** gender equality, gender-sensitive design, culturally transformative design, critical reflexivity, queer phenomenology, negative capability

## Abstract

Design, as a practice of developing solutions beyond products, and increasingly services and policies, inevitably poses an impact on gender (in)equality which remains largely unrecognized by design practitioners. This paper advocates the urgent need for adopting gender lenses in design education for sustainable cultural transformation through proper recognition of the complexity of any societal and cultural issue, power relations and inequalities, and introduces an initial attempt through a graduate-level educational design project. Throughout the project, students critically reflected on existing orientations in designing to develop norm-critical gender lenses, contained the resultant disorientation emerging from the contrast between their critical approaches and local contexts, and explored novel directions as reorientation to address four different societal and cultural issues and develop 11 design outcomes aiming at gender equality, social justice-oriented empowerment, and cultural transformation. The authors analyzed the design processes and outcomes to reveal opportunities and challenges for developing and deploying norm-critical gender lenses in tackling complex, intersecting socio-cultural and political issues, under three themes: gender stereotypes, norms, expectations, and roles; intersectional power relations and inequalities embedded in the social structure; and social justice-oriented empowerment beyond the market-oriented individualistic neoliberal order. A shift in the perceptions of the role of designers, from creator/problem-solver to facilitator/participant, and design outcomes, from absolute solutions to intermediaries of sociological and political imaginations, is found crucial in this endeavor, which requires safe spaces for future designers to reflect on existing orientations, contain disorientation with negative capability, and explore novel ways through reorientation.

## 1 Introduction

Gender inequality and its detrimental impact on human rights are actively communicated at the global/policy level through the Sustainable Development Goals and EU’s Gender Equality Strategy. Gender mainstreaming has become acknowledged as a strategy to overcome gender inequality as part of sustainable cultural transformation in recent years, through its recognition as a cross-cutting issue in all aspects of cultural and social life. As an intervention area in all aspects of life, gender is recognized as an analysis category revealing otherwise implicit sources of inequality (Scott, 2010). As a practice of developing solutions beyond products and technologies, and increasingly services and policies, design inevitably has an impact on gender (in)equality. This impact, however, remains largely unrecognized by practitioners lacking the necessary knowledge, methods, or tools to address such a complex, multi-layered, socio-political concept. Gender as an integral aspect of design problems and solutions finds little mention in design education and mainstream design practice, where gender stereotypes and gender blindness proliferate. Gender issues in higher education generally, or design education specifically, are explored in terms of representation or task allocation (Kiernan et al., 2023) and mostly as a variable but not as one of the fundamental organizing principles of society (Benhabib, 2002; Henderson, 2018). There remains a gap in how, actually, norm-critical gender lenses can be fostered in future design professionals to inform their future design practice, especially considering the ever-widening scope of the profession and the wide-ranging societal impacts of its outcomes. It is imperative to continuously reflect on the ways design choices reinforce or resist certain norms of gender, sexuality, race, class, and ability, and affect the lives and experiences of marginalized groups throughout the design process. However, design curricula or academia do not inherently incorporate gender-related issues and fall short of facilitating such reflection.

This paper advocates the urgent need for adopting gender lenses in design (education) for sustainable cultural transformation through proper recognition of the complexity of any societal and cultural issue, power relations and inequalities. However, design students and practitioners are mostly oriented toward rather abstract notions of innovation and creativity which are prone to ignore the complexity of societal and cultural issues, power relations and inequalities, and also risk reinforcing existing gender norms and stereotypes and drift apart from social justice-oriented empowerment. This paper demonstrates that developing innovative and creative design outcomes addressing such complex societal issues is not a straightforward endeavor but requires a complex pedagogical process involving detours. Building on Ahmed’s (2006) queer phenomenology, nourishing norm-critical gender lenses primarily involves providing a safe space that allows the students (a) to critically reflect on existing orientations of designers and users toward social objects in the forms of gendered norms and practices, (b) to acknowledge and embrace emerging disorientation by developing the negative capability to contain varying uncertainties and uneasiness, and (c) to explore novel directions and reorientate toward social justice oriented empowerment and cultural transformation throughout the design process. This process also requires design students’ recognition of and engagement with the context of design interventions, including the existing local policies and practices as well as the specific contextual needs, opportunities and challenges of different communities, so that these interventions can potentially initiate cultural transformation for gender equality (GE) by addressing gender issues in more concrete and realistic ways. Such a process is, however, challenged by the widely adopted problem-solver and outcome-oriented approach in design, which can result in abandoning the critical reflexivity on existing orientations and negative capability to contain disorientations during the process. This approach inherently leads to heuristically returning to gendered norms and expectations, and entrenching unequal intersectional power relations that the design process aimed to address in the first place. Instead, we argue that design outcomes should instigate empowerment from within and should be regarded as intermediaries of sociological and political imaginations rather than absolute solutions.

In an attempt at partially closing this gap, the authors (i.e., a gender scholar and a design educator) developed a graduate-level educational design project in collaboration with Çankaya GE Unit. In the following lines, we first present the background of this study and then explain the project structure and methodology we deployed. As part of the project, four different cultural and societal issues on gender inequality in Ankara emerged from the dialogs and collaborations among the students, the authors and the Çankaya GE Unit, and 11 design interventions were developed. We briefly introduce these design processes and outcomes, and continue with their analysis to reveal the opportunities and challenges of design practice and education in addressing (a) culturally accepted and embodied gendered stereotypes, norms, expectations, and roles, (b) gendered aspects of power and inequalities embedded in the social and cultural structure, and (c) empowerment aiming at GE and justice beyond the limited and misguided understanding of market-oriented, individualistic neoliberal order. Finally, we discuss these beyond the scope of this educational case to inform design education and design practice settings in fostering, adopting, and deploying norm-critical gender lenses, which inevitably harbor disorientation and reorientation throughout the design process. We argue that critically reflecting on existing orientations, embracing disorientation with all its uneasiness and uncertainties, and exploring novel ways for reorientation in the design process is crucial, especially for design educators, students and practitioners with similar concerns and interests to develop critical reflexivity and innovative design outcomes that initiate cultural transformation in favor of GE.

## 2 Background

### 2.1 Gendered norms, stereotypes, and bias in design

Gendered norms and the resultant gendered stereotypes, expectations and roles are widespread, diffused and implicit in everyday life. Gendered stereotypes for women involve the roles of mother and wife, limited to their caregiver and nurturer duties and being dependent and complacent which are associated with their ascribed roles in reproduction and mostly as part of their unpaid emotional labor (Hochschild, 1983). For men, they involve the roles of breadwinner, provider, achiever and being self-sufficient, competitive and autonomous which are seen as essential features for their ascribed roles in production within the capitalist market economy. These stereotypes entrench gendered roles and expectations in people’s social and professional lives even further, especially limiting women’s capabilities (Eccles, 1987; Heilman, 2001), and mostly pose a conflict to their both practical and strategic gender interests. For example, women are mostly expected or consigned to take on occupations that involve emotional labour and care work or to work at home unpaid such as caring for children and the elderly. Besides that, the discrepancy between these socially ascribed gendered roles/expectations and gender needs dramatically deepens, especially when gender-blind social policies prevail (Criado-Perez, 2019). Such policies are characterized by the unrecognition or misrecognition of different needs and interests of varying gendered subject positions for their wellbeing. However, addressing this discrepancy is challenging, since gendered roles and expectations are ingrained in societal perceptions as well as in people’s perceptions of themselves, and despite contradicting gender needs and interests, people are oriented toward such norms that evoke feelings of comfort and being at home (Ahmed, 2006). Those who fail to meet these expectations are stigmatised by society, potentially resulting in feelings of frustration, failure and being disoriented. Considering how prevalent these roles and expectations are at the individual and societal levels, roles/expectations and gender needs cannot be untangled smoothly in practice without developing a critical reflection of both designers’ own and users’ existing orientations toward gendered norms and expectations.

While the past couple of decades witnessed various global-level improvements in amending social policies in this regard and toward GE through a sustainable cultural transformation, these efforts still remain within the hetero-patriarchal gender binary and in consideration of idealised masculine and feminine norms and expectations. Hence, LGBTQ+ and others not conforming to the gender binary are excluded from these efforts and renewed policies remain gender-blind to their diverse practical gender needs and interests, let alone more strategic, long-term interests (Molyneux, 1984; Moser, 1993). Furthermore, there is an ongoing counter-process highlighted within anti-gender politics and rhetoric undermining varied gender interests and strengthening traditional gender stereotypes and expectations while criminalizing the existence of LGBTQ+ beyond the un/misrecognition of their needs and rights. This is also evident in Turkey,^1^ where the discrepancy between the traditional gender roles/expectations and gender needs is growing more (Yetiş and Kolluoğlu, 2022). This counter-process increasingly hinders the development of such necessary critical reflexivity in practice.

While there is a growing corpus of research and practice in technology-related fields in favor of GE, there is also a need to recognize the impeding impact of anti-gender politics at both local and global scales, both for now and in the future. Parallel to such tension, gender bias in technology-related fields persists despite efforts to eliminate it (Lechman and Popowska, 2022), which can be attributed to the limited adoption and development of norm-critical perspectives as well as the practitioners’ limited reflection on how they are oriented toward such norms and biases in the first place. When it comes to design, gender stereotyping is at work in dichotomous relation between certain sub-fields of design that are considered suitable for men and others for women (Kaygan, 2016). This separation is also apparent in task allocation throughout the design process, in which more technological, technical, “hard” design tasks are attributed to men (Kaygan, 2014). The design outcomes also reproduce gender stereotypes by reinforcing the ascribed relationship between certain esthetic/functional features and gender via reflecting the gendered division of domestic tasks, e.g., washing machine as feminine due to washing clothes is considered a women’s task (Aaltojärvi, 2012) or hegemonic gendered relations common in work settings, e.g., sophisticated and prototypical fountain pen designs reflecting business masculinity associated with executive men (Kaygan et al., 2019). Such design practices end up reinforcing hegemonic gender order grounded to the gender binary, and create further barriers and intensify exclusionary repercussions on gender non-conforming others on the individual scale (Denz and Eggink, 2019). When designing for/with communities, the guidelines on forms of communication, collaboration and engagement as well as the moderation of participation, accommodate certain individuals and communities while implicitly or inadvertently excluding others (Denz and Eggink, 2019; Costanza-Chock, 2020). Finally at the institutional scale, designing services and systems almost always involves various forms of prioritization stemming from funding resources and policies and leads to the development of services and systems hyper-focussed on those priorities, which end up continuously redistributing benefits for the privileged and harms for others (Costanza-Chock, 2020). This unjust redistribution exacerbates social harms by adding up to the cumulative disempowerment of disadvantaged, marginalized individuals within the existing hegemonic gender order. However, design can also challenge gender norms by being more inclusive and diverse, and by considering the needs and preferences of different users, not just the dominant or stereotypical ones within the gender binary (Ehrnberger et al., 2012; Denz and Eggink, 2019; Costanza-Chock, 2020). To achieve that, gender-sensitive design processes and practices should be developed to address the current discrepancy between gender norms/expectations and gender needs by analytically and realistically differentiating these and providing solutions accordingly. However, this is also a challenge for design practitioners that require reflexive evaluation of both the gendered aspects of the design process and their own attitudes toward, and embodiment of, these already gendered practices which are imbued through their individual socialization as well as their acquisition of designer roles and practices.

### 2.2 Intersectional systems of oppression, hegemonic gender relations and design

Gender studies literature provides us with a large breadth of approaches, as well as empirical data, for building gender lenses and the capability to recognize these endless forms of discrimination, exclusion, exploitation, domination, and violence in all aspects of life. The conceptualization of gender does not only indicate the ground social injustices build upon but also provides an analytical tool to comprehend, reveal and eliminate them (Scott, 2010). Gender, as a concept and as an analysis category, crosscuts all domains involving power relations and poses opportunities for revealing various issues of power and inequalities embedded in the social structure. Thus, gender inequality cannot be addressed as a standalone societal issue with clearly defined borders, like any other exclusionary, discriminatory, oppressive issues; they almost always intersect and compound various forms of inequality in individuals’ lives (Crenshaw, 1991). This does not result in a straightforward sum of experienced discriminatory practices due to gender, sexuality, age, race, class, dis/ability, etc., but generates more complex, intertwined obstacles that are harder to recognize, dismantle and respond to beyond such categorizations (Nash, 2016). Hence, designers must recognize gender not as an exclusive domain of application mostly encompassing women’s issues, but as a lens for analyzing and revealing varied power relations and injustices within all domains of life. In this regard, the intersectionality approach (Crenshaw, 1991; Costanza-Chock, 2021) proves especially useful, as it highlights how different forms of inequality and oppression are experienced by different intersectional positions and identities.

The intersectionality approach needs to go beyond finer, universal categorizations better identifying distinct marginalized groups through intersections of various sources of inequality and to involve the capability to recognize how these intersect, compound and are embodied in endless forms, uniquely affecting individuals in different contexts. While distinctions that separate certain groups from others can be utilised for advocacy by making discriminatory practices visible and claiming rights, a cultural transformation for non-discrimination, anti-oppression and social inclusion is needed through critically questioning the positions of the privileged considered as hierarchically “higher” and as a norm (i.e., *cis*, heterosexual, and white man), empowering disadvantaged individuals and communities, and realizing rights as part of the response to social injustice arising from structures of power and domination at multiple scales (Cornwall and Rivas, 2015). This requires a critical examination of the existing gender regime consisting of numerous interlocking systems of oppression such as patriarchy, racism, and capitalism (Hill Collins, 2000).

Critical masculinity studies also discuss the hierarchical relations between men and women and among men, and how such relations create and justify unequal practices, obstacles and disadvantages for women as well as subordinate and marginalized men in relation to hegemonic masculinity (Connell and Messerschmidt, 2005). However, hegemonic masculinity and its ascribed features are not static or pre-given as they are re-articulated and sedimented according to political and historical contexts (Hultman and Pulé, 2019). Even if it is an “ideal” depiction of features that no man ever fully possesses, it assures the discursive and material superiority and domination of men over women and marginalized/subordinate men (Childs and Hughes, 2018). As such, the existing gender regime creates obstacles and disadvantages not just for women and LGBTQ+, but also for *cis*, heterosexual men, e.g., young, with disabilities, from disadvantaged ethnic minorities or lower socio-economic backgrounds. What makes this “hegemonic” cannot be reduced to the enactment of power by force and violence *per se*, but involves the processes of consent creation to sustain and legitimise its gendered meaning of power, authority and privilege through existing gendered stereotypes, norms and beliefs. Hence, developing the capacity to recognise, critically analyse and dismantle these processes of legitimization and consent creation is crucial for lasting cultural transformation. In this regard, there is a need to focus on the role of design and technology not only in ensuring that the design of products and services does not reproduce and strengthen existing intersectional systems of oppression but also in dismantling them and re-imagining non-exclusionary, anti-oppressive, empowering alternatives (Andersson et al., 2017; Costanza-Chock, 2020). This requires both the deployment of critical approaches and a new safe space for design that can accommodate critical thinking and practice beyond the hegemonic gender order and counter-hegemonic practices both for design practitioners and beneficiaries of the design outcomes. However, since such a space for design cannot be realized outside the reality of the existing hegemonic order, these outcomes could be much harder to implement. Thus, the whole endeavor to build such a space for nourishing these potentially counter-hegemonic practices is an initial and essential goal by itself. This endeavor should involve not only a space of critical self-reflection on existing orientations within the systems of oppression but also the development of a negative capability to contain resulting uncertainties and uneasiness (experienced as disorientation). This should be regarded as an essential part of redefining social justice-oriented empowerment that paves the way toward novel directions for design practice.

### 2.3 Meaning of empowerment for design

Empowerment, as a concept standing for achieving social justice, equity, and equality, requires social action, collective effort, and participation (Thompson, 2007). However, it has begun to be perceived and marketed at a very individual level with a neoliberal interpretation, and its focus is being reduced from the transformation of the social structure in favor of social justice to a list of personal decisions and achievements that the individual can autonomously take (Cornwall and Rivas, 2015). Instead of developing an appropriate strategy of empowerment to correct the concrete negative conditions that generate social exclusion, discrimination, injustice and inequality and change the system that produces these conditions, a narrative of empowerment is marketed and promoted consisting of a few individuals’ achievements as a result of their personal struggle against their own negative conditions throughout their life courses. While individuals’ wellbeing and achievements are important by themselves, the empowerment of individuals cannot be interpreted as real empowerment if it is only achieved at the expense of the whole community. This is another place where design can become complicit in reproducing the matrix of domination, as a practice of generating means for individuals only to *adjust* to and *make do* within the existing social structure.

Such individualistic empowerment narratives are emanated from the socio-economic development agenda of Global North and then promoted to the rest of the world within the package of universal principles of the neoliberal market economy. Beyond their rationale, such narratives bring forward affective rhetoric based on being passionate, dedicated, motivated, and having an individual entrepreneurial spirit. These reduce individual empowerment to having certain affective qualities by ignoring the material realities and conditions which include the multitude of other structural barriers against such individual empowerment that said affective qualities are not enough to overcome. Let alone encouraging people, such affective rhetoric and individualistic success narratives become exclusionary by themselves (Harvey and Shepherd, 2016). This exclusion is based on ignoring not only the material realities and conditions but also the overwhelming, negative emotions and affections caused by these disempowering conditions—which may even end up reinforcing feelings of frustration, helplessness, and a sense of inadequacy for people who are already disoriented within the existing system. Designers need to contemplate the meaning of the affective dimensions with negative capability^2^ as an essential part of empowerment at both the individual and community scales, as well as their own capacities for such empowerment aiming at inclusivity during their design practices—in which reflexivity on dominant orientations and assumptions, such as heteronormativity, colonialism, capitalism, and ableism, becomes crucial once again.

The relationship between designing and empowerment cannot be interpreted as the designer providing means for others’ empowerment as innovative design outcomes do not necessarily result in empowerment without a wider gender transformative agenda (Brugere et al., 2020). It should rather be interpreted as the empowerment of communities, including the designers, through more participatory and inclusive efforts. Participation and inclusivity are increasingly discussed in design literature, and there are various design frameworks that aim to empower users who face different forms of marginalization. For example, inclusive design was originally developed to address the needs of people with disabilities but now tries to address a wider range of factors that can lead to exclusion, such as gender and socio-economic status (Szlavi and Guedes, 2023). Participatory design involves users as co-designers and co-creators of design solutions, giving them a voice and a stake in the design process, and more recently began to tackle gender equality issues (Iivari et al., 2023). Design for social innovation (Manzini, 2015) and autonomous design (Escobar, 2018) are perhaps the most relevant frameworks, as they focus on empowering people at the local level through collaborative design activities. These activities can have various benefits for the participants, creating employment opportunities, enhancing social participation, building community bonds, fostering solidarity, achieving self-actualization, and improving overall wellbeing. However, these frameworks do not necessarily have a specific focus on gender equality or a clear way of measuring their impact, except for the more recent design justice framework (Costanza-Chock, 2021). Designers’ awareness of values on gender and recognition of the potential consequences of their designs remains crucial for gender-sensitive design practices (Rommes, 2014). How do design choices reinforce or resist certain norms of gender, sexuality, race, class, and ability? How do design outcomes affect the lives and experiences of marginalised groups? How can design be more inclusive and diverse? Designing for social justice-oriented empowerment requires continuously asking these questions throughout the design process, yet gender-related issues aren’t inherently integrated into design curricula or academia, which fall short of facilitating such reflection.

### 2.4 Gender-sensitive design in the context of Turkey

The educational design projects introduced in this paper were carried out in Çankaya, Ankara, Turkey, where the local municipality has been a pioneer in promoting GE and women’s rights in its services and projects. The municipality signed the European Charter for Equality of Women and Men in Local Life in 2013 and implemented the Local Equality Action Plan to ensure GE in its policies and practices. Çankaya GE Unit aims to initiate lasting cultural/institutional transformation through mainstreaming gender and collaborates with external actors (e.g., CSOs, universities) to carry out impactful projects and address gender issues in Çankaya and Ankara. However, the municipality has been experiencing a sort of disorientation, facing obstruction and conflict with anti-gender politics underpinned by the central government, such as restricting women’s access to abortion and contraception, promoting traditional gender roles, excluding women from decision-making mechanisms regarding both their own gender interests and politics in general, and withdrawing from the Istanbul Convention on preventing and combating violence against women and domestic violence on the grounds that its anti-discriminatory clauses damage the traditional family values (Yetiş and Kolluoğlu, 2022). The anti-gender rhetoric of the ruling party poses women and LGBTQ+ movements as a threat to the social order and national identity of Turkey by associating these with Western values and influence. This is also evidenced in the increasing tendency for renaming gender and women’s studies centers as family and women studies centers, and the removal of “gender” from the names of some university courses after the Higher Education Authority revoked its policy on GE in 2019 by condemning the very concept “gender” as inappropriate to societal norms and values (Uçan Çubukçu, 2021). Furthermore, LGBTQ+ assemblies, including but not limited to the Pride Parades, have been banned every year for the past decade, on the grounds that these events “threaten the institution of family” (Human Rights Watch, 2023). This anti-gender rhetoric entrenches the existing socio-political polarization even in mundane events and results in its reception at varying degrees in society (Ozduzen and Korkut, 2020). However, this does not indicate substantial anti-gender movements impacting government policies from the bottom-up; rather, it indicates top-down anti-gender politics^3^ and rhetoric affecting public opinion (Yetiş, 2023), as evidenced by women-led GONGOs (government-supported non-governmental organizations) emerging over the past couple of decades (Ehrhart, 2022). Hence, top-down anti-gender politics spearheaded by the ruling government does not necessarily mean there is a widespread anti-gender movement backed by society in general in Turkey (Yetiş and Kolluoğlu, 2022). Such misconception would result in a pessimistic view and hinder exploring alternatives.

Although anti-gender politics is a global issue warranting a global struggle, it is not realistic to apply a generalized remedy without recognizing national and local gender regimes and power relations. This is also similar for the global GE agenda spearheaded by Global North packaged as part of development goals, which does not necessarily lead toward cultural transformation in the rest of the world directly even when such policies are adopted by countries on paper (Goetz, 2019). No matter the commitments signed, strategic gender interests remain abstract and out of touch so long as these changes are hindered, or even challenged, by local communities in the realization of intended changes. This brings forward the importance of local policies and their implementation to achieve GE. While its efforts are continuously being thwarted by the central government, the Çankaya Municipality attempts to address gender inequality with local projects and programs supporting women and LGBTQ+ (SPoD, 2019; Şener and İnanç, 2021). In our collaboration with the Çankaya GE Unit, we brought forward that design can help develop different strategies and policies by creating novel ideas that can be implemented with the limited resources and legal capacity of the municipality. Accordingly, the educational design projects presented in this paper aimed to explore how adopting gender-sensitive lenses in design projects can address the local GE issues in Çankaya, as well as the challenges and opportunities of conducting such a project in a socio-political context where anti-gender politics are enacted by the central government. Focusing on local can be considered as both a strategy and a preference for GE and empowerment, as it allows identifying and addressing the practical and strategic gender needs relationally. In some cases, depending on the dynamics of the community, cultural transformation from local can be more feasible and effective than widespread national-level transformation, especially when varied obstacles are posed by the central government. Such local achievements in GE and empowerment can be exemplary and transferrable for other local communities and create a cascading effect in turn. The authors believe such local, grassroots interventions can result in more grounded, profound and realistic achievements of cultural transformation in favor of GE. In this regard, the goals of the GE Unit and the authors were aligned in recognition of the local dynamics for such transformation. To this end, the design processes and outcomes of this education project emphasized the importance of local GE policies and their implementations, considering the varied and intersectional gender interests and contributing to social justice-oriented empowerment.

## 3 Methodology

This study involves the development and implementation of an educational design project in an attempt to build graduate students’ knowledge, skills and capabilities in deploying norm-critical gender lenses in their design practice and questions how these perspectives are perceived, received and implemented in design processes by students, to explore the challenges and opportunities observed by the authors, external experts, and the students. The process involved working on the problems the authors identified, continuous self-evaluation, and bringing various stakeholders (e.g., instructors, researchers, and collaborators) together to improve pedagogical practice and contribute to educational theory with an action research approach (Oja and Smulyan, 1989; McKernan, 2007). Accordingly, the authors first developed the theory-informed project structure and syllabus^4^ in collaboration with the Çankaya GE Unit (Stage 1 Planning), deployed this structure in the Graduate Design Studio course (Stage 2 Action), and analyzed the design processes and outcomes (Stage 3 Analysis).^5^

**Figure 1:**
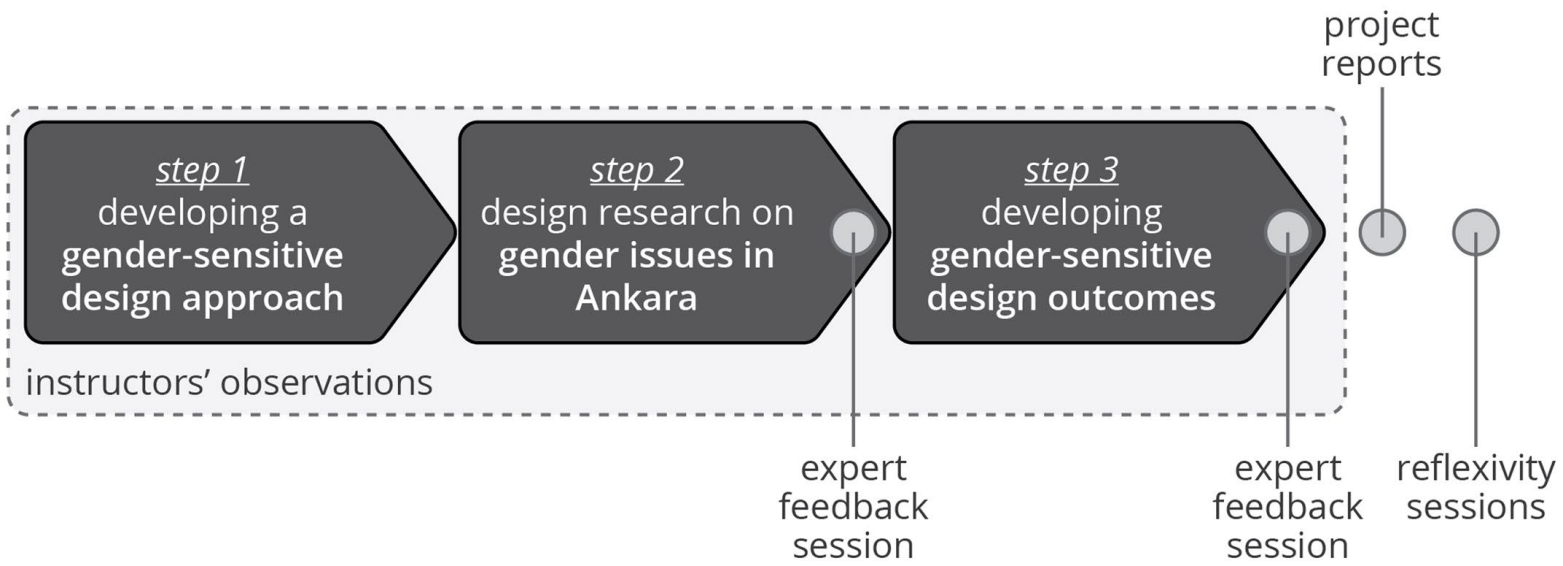
The project structure of the educational design project.

The students followed the same project structure consisting of three steps (Figure 1); however, they focused on different societal issues. The *first step* involved a literature review on distinct yet interrelated topics, such as the transformative approach to GE, empowerment, gender mainstreaming and intersectionality; sustainability and environmental crisis through a gender lens; bodies and objects within a gender perspective through object-oriented feminism, queer phenomenology, affect theory, social innovation, pluriverse and southern theory. The purpose here was three-fold: (a) to gain a comprehensive understanding of the concept of “gender” and how it crosscuts every social and cultural issue, as well as its implications for the design process and outcomes, (b) to critically explore and contemplate designers’ changing roles and capabilities, and (c) to build a critical socio-political and socio-cultural perspective through contemporary gender theories and feminist philosophy. At the end of this step, the students have formed groups (three groups with three members and one group with two members) and decided on broadly defined scopes each team will explore in the following stages. They decided on these scopes themselves, through class discussions and feedback from the instructors and Çankaya GE Unit. In the *second step*, the students deployed this critical perspective during the design research stage, using methods like design ethnography, interviews with experts and front-line workers, surveys and/or generative design research tools, identified specific problem areas to focus on, and developed specific design briefs. *This design research step should not be confused with the educational action research methodology deployed by the authors; it refers to a step in the design process performed by design students*. The *third step* involved the development of design interventions for these problem areas in Ankara on selected societal issues (i.e., scopes). The number of design interventions developed was decided based on the number of team members (e.g., 3-member teams three design interventions).

This structure was deployed in the Graduate Design Studio course at Middle East Technical University, Department of Industrial Design under the supervision of two instructors (a sociologist/gender expert and a design researcher/educator), in collaboration with Çankaya GE Unit. This was a 14-week course with two sessions every week (i.e., 9 h every week), and 11 graduate-level design students took this course. The rather limited time (~4 months) of the course inevitably affected the overall quality and level of detail of design outcomes. The design processes and design outcomes focused on four different socio-cultural and political issues. This variety was also reflected in the gender lenses the students developed, the research methods they deployed and the interventions they designed throughout the project. The authors deployed the triangulation of data collection (Piggot-Irvine, 2008) with four sources of data (light gray in Figure 1) generated throughout the educational project to analyze the opportunities and limitations of fostering norm-critical gender lenses in future design professionals and to ensure that the analysis reveals reliable and meaningful results:

(1) *instructors’ observations*, recorded as field notes during classes, regarding the challenges and opportunities of developing and deploying gender lenses throughout the design process for a cultural transformation,
(2) *two structured feedback sessions with experts* (a service designer, a design researcher with expertise in material culture, an STS researcher and expert on gender in STEM, and two municipality officers), revealing varying concerns, omissions, relevance, and possible impact of projects,
(3) *students’ project reports* prepared at the end of the projects, revealing decision-making processes at different stages,
(4) *reflexivity sessions with graduate students*, contemplating on our and their positionality in terms of power relations and professional authority/capability, challenging/empowering aspects of adopting gender lenses, gendered subjectivities as embodiment, and affective dimensions of their design thinking, processes and outcomes (Keddie et al., 2021).

These data sources were analyzed in a complementary manner, to identify similarities and differences. The feedback sessions with external experts were used to reduce observer bias. The process and outcomes were analyzed in terms of how the students perceived, received and implemented the gender lenses through the critical perspective they tried to develop. The purpose of the analysis was not to assess the applicability or effectiveness of these projects but rather to explore orientations within the existing contexts, disorientations as challenges encountered during the design process and reorientations as novel directions for design practice with critical reflexivity and innovative outcomes.

## 4 Design outcomes deploying a gender-sensitive approach in the context of Turkey

This section briefly introduces the four societal issues addressed and 11 design outcomes developed by the students. Table 1 presents these four scopes, in terms of larger societal issues selected, student team formations, design research methods deployed, main concerns identified, and design outcomes developed. This is followed by brief explanations of the design outcomes for each scope, to contextualize the analysis in the following section.

**Table 1:** The scopes, main concerns, research methods, and outcomes of projects.

	Scope 1 (S1)	Scope 2 (S2)	Scope 3 (S3)	Scope 4 (S4)
Societal issues	Gender and mobility/transportation	Women empowerment	NEETs and social injustice	Gender awareness
Students	Two students, with design backgrounds	Three students, with design and engineering backgrounds	Three students, with design and education backgrounds	Three students, with design and visual communication backgrounds
Design research methods students used	Survey (*n* = 72, 45 women and 27 men), semi-structured interviews (*n* = 7, 5 women and 2 men)	Semi-structured interviews (*n* = 13), social media analysis	Survey (*n* = 36), semi-structured interviews (*n* = 10)	Semi-structured interviews, generative design research tools (*n* = 13)
Main concerns	Gendered sense of safety, gender stereotypes, stigmatisation based on age, ethnicity, dis/abilities, gender expression, etc.	Individual and community-based empowerment in women’s communities, with a specific focus on Covid-19 impact	Gender stereotyping and stigmatisation of NEETs, “maintenance” as inertia, NEETs’ participation in public sphere	Gender roles and stereotypes in relationships (private, social and professional), everyday objects and daily practices
Outcomes	Two outcomes: monitoring system for busses & a mobile application for transport; a bus stop design	Three outcomes: accessory set for women’s community; training and collaboration project; an online sale stand platform	Three outcomes: consultancy service entry point; social media app; third space and support app	Three outcomes: three distinct generative tools for gender education and training

**Figure 2:**
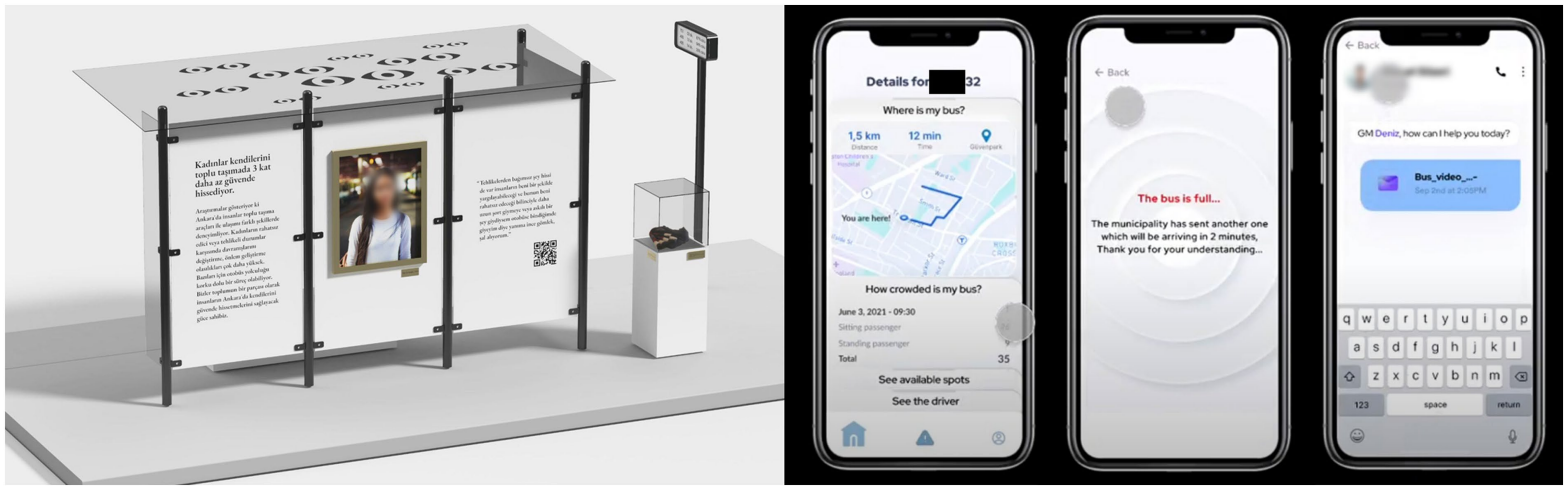
“Bus Stop Museum” (left) and screenshots from the mobile app of the in-transit spatial awareness system showing the interfaces of a specific bus route, occupancy alert for a specific bus and customer service chat box (right), by Yaren Palamut and Max Plummer.

The S1 focused on gendered aspects of mobility and developing awareness of both social and spatial aspects of bus experience (Figure 2). *Bus Stop Museums* aims to make the gendered sense of safety and gender inequality in mobility visible through relevant statistics for Ankara, real-life concerns as quotes, the pedestal of various additional objects (e.g., the additional t-shirts, scarves) women utilize just to avert unwanted public attention mostly regarded as harassment. *In-transit Spatial Awareness System* is an improved occupancy sensor system coupled with driver interfaces for risk assessment, an app for commuters to track the occupancy status of busses and make informed decisions about their commute, and a set of procedures involving other relevant stakeholders (e.g., law enforcement) and the extent of data (not) to be shared in case incidents occur.

**Figure 3:**
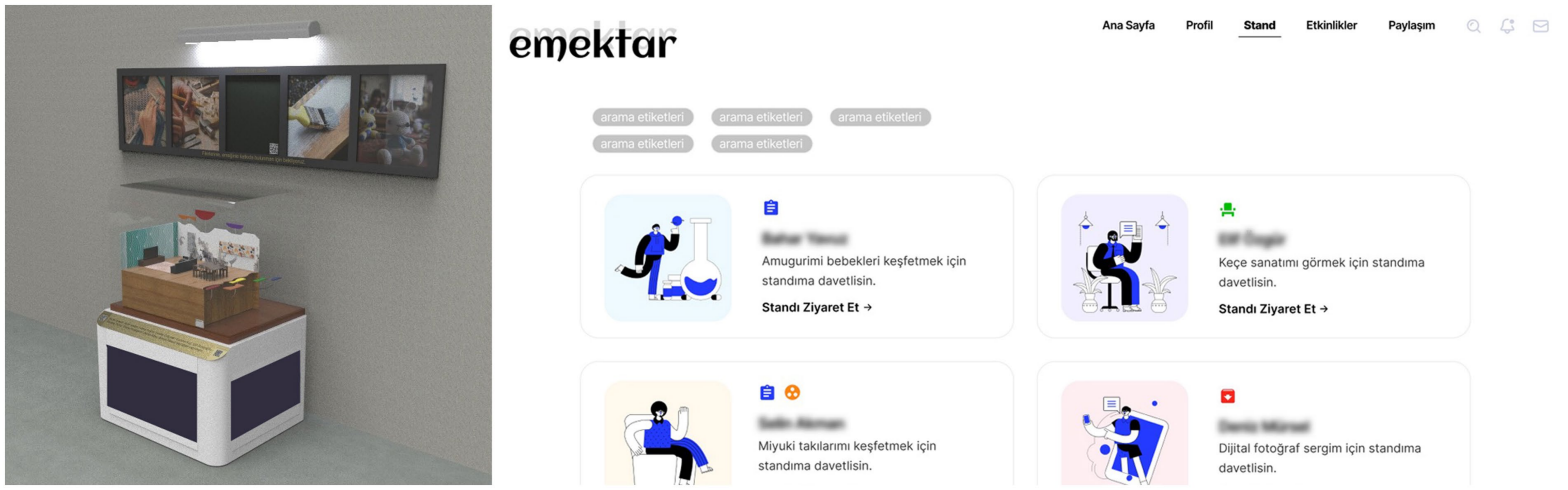
“Production, Together” stand for women’s production communities provided by Çankaya Municipality (left) and a snapshot of “Emektar” online sales channel that lists the “online stands” for limited-time sales events (right), by Enes Coşkun, Eren Dönertaş, and Sümeyye Şimşekler.

S2 focused on women’s communities supported by Çankaya Municipality through providing vocational training and sales channels for the products they produce and developed designs for further individual and community-based empowerment (Figure 3). *Invisible Labor Kits* consist of craft materials for, e.g., accessory design, embroidery, publishing, etc. and codify forms of care and emotional labor in daily life. The community members can bring together codified materials to visualize their invisible labor and recognize both the shared and differing practices among the community members. *Production, Together* involves community members deciding on a theme, code signing and producing an object/scene to be exhibited along with a narrative of the codesign process, in a space maintained by the municipality for a limited time, and then online auctioning of the coproduced objects to fund the next community project. *Emektar [Turkish noun for age-old and loyal worker]* is an online knowledge-sharing and virtual sale stands platform that hosts online sales events regularly, which can create a call for solidarity and support through enhanced reach and attraction of a larger audience.

**Figure 4:**
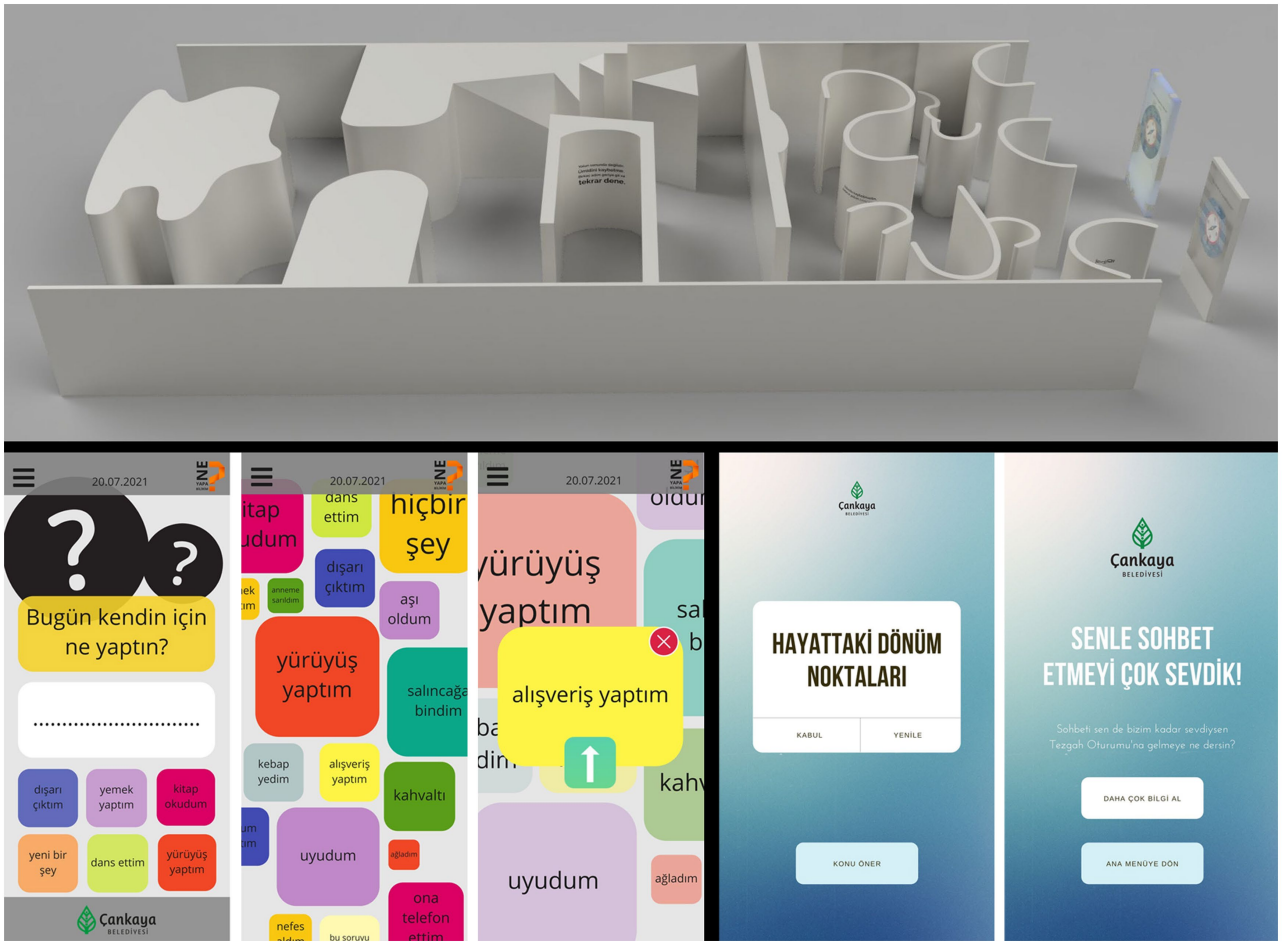
Details from “Labyrinth/Compass” installation (~8.5 m × 16 m) designed for Kızılay Metro Station (upper), screenshots from “Grimap” social sharing platform (lower left), and screenshots from “Tezgah” topic suggestion app for initiating conversations (lower right), by İlayda Karadeniz, Zeynep Özcan, and Meryem Özkan.

S3 focused on NEETs (Not in Education, Employment, or Training), explored the social injustice they experience through the perspective of slow violence (Nixon, 2013; Yetiş and Bakırlıoğlu, 2023) and developed three design interventions (Figure 4). *Labyrinth*/*Compass* is a consultancy service entry point for NEETs who would not normally engage with such services, involving a labyrinth-like setup placed in commonly used public spaces, depicting feelings of lostness, disorientation, hopelessness, and injustice by highlighting gendered paths leading to different outcomes between men and women. *Grimap* is a social media application for people stuck at home, accessible through QR codes on everyday items like plastic bags and packaging and encourages them to socialize beyond their families and connect with other NEETs by sharing their daily routines. The application aggregates similar practices that are different from working or studying individuals, to visualize that these people aren’t actually alone in doing them at similar times. *Tezgah [Stall]* is about creating a counter-hegemonic “third place” (Oldenburg, 1989), as a safe space, for NEETs where they can engage in the co-creation of anything, such as conversations, discussions, ideas, new ventures, and novel ways of interaction (Yetiş and Bakırlıoğlu, 2015). Since initiating such social interactions is a challenge among people who leave their comfort zones and meet for the first time, it involves a supporting application for conversation starters suggesting various topics based on NEETs’ shared experiences in Ankara as well as details for moderation.

**Figure 5:**
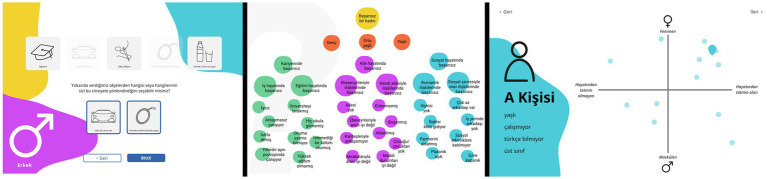
Screenshots of gender awareness training tools “Randomizer,” “HowUFail,” and “PinMap” (left-to-right) developed by Elif Dilara Bora, Açelya Küçükkurt, and Öyku Elif Şare.

S4 focused on improving gender awareness in Çankaya and developed three different generative tools for gender education and training with potential to go beyond unilateral, didactic and passive learner approaches to education and deploy more interactive, open-ended and norm-critical approaches (Figure 5). The tools were designed for potential gender awareness training programs. *Randomizer* randomly brings a set of five objects and asks the trainees the gender of the person who would have those and which objects made them think so, with an algorithm that refrains from creating combinations oriented toward any existing gender stereotypes. *PinMap* proposes different characters based on intersectionality according to criteria identified by trainers and asks the trainees to pin these characters on a coordinate scale of their gender expression (feminine to masculine) and their satisfaction in life (content to not content), to reveal the existing orientations of trainees toward certain social objects. *HowUFail* challenges heteronormative gender stereotypes attributed to success and invites trainees to redefine what can make a person “successful” in novel ways other than these gendered norms and stereotypes, adopting Halberstam’s (2011) queer art of failure^6^ that regards failure as a productive way of critiquing capitalism and heteronormativity, which can help trainees to develop the negative capability to recognize and contain disorientation in an affirmative way.

## 5 Opportunities and challenges of disorientation and reorientation in design education

The analysis of these design processes and outcomes revealed various opportunities and challenges at different steps of the project. In this section, these are presented under three prominent and interrelated themes: (1) addressing gender stereotypes, norms, expectations, and roles, (2) addressing the intersectional power relations and inequality embedded in the social and cultural structure, and (3) addressing social justice-oriented empowerment beyond the market-oriented individualistic neoliberal order.

### 5.1 Addressing gender stereotypes, norms, expectations, and roles

Gender stereotypes, norms, expectations, and roles must be recognized and critically dismantled to understand multi-faceted societal issues (i.e., the scopes students identified) in terms of different needs as well as to address these issues through design with long-term, strategic gender interests formulated as critical, inclusive GE agenda. These roles are replicated and cascade even in activities that aim at empowerment and challenging existing gender norms. For example, design research on women’s communities’ sales events (S2) revealed that many husbands were supporting their partners’ business activities; however, many of them handled the money exchange and register—pointing to yet another gendered role. This contrasts with women’s capacities for management, evidenced especially through the creative management of the household domestic economy, and reproduces the stereotypical family man that handles the money in the public sphere (Johnson, 2012).

Exploration of all four societal issues involved the recognition of people experiencing all aspects of life as gendered subjectivities, and gendered norms and expectations being continuously imposed on everyone. S3 illustrates this clearly, in which students found that although the statistics about NEETs were almost always broken down to women and men, the numbers were inconsequential in explaining obvious differences between varied gendered subject positions of NEETs in certain aspects. The team initially approached gender issues as a substitute for the term women’s issues (Smith, 2019) and disregarded other gendered subjectivities. However, their design research further clarified that being NEET is experienced completely differently by women and men due to gendered norms and expectations, in relation to their class and age, imposed on them. It also revealed that being NEET means something entirely different for gender non-conforming people since they already face barriers even entering work, life, and education in the form of further marginalization and social exclusion (Bradlow et al., 2020). NEETs adopted “maintenance” (Baraitser, 2015) practices—not as doing *something*, but as not-doing and embracing inertia as a lifestyle—to endure the impact of lifelong gender stereotyping and stigmatization imposed by society. While men are only expected to be successful, competitive, achiever, and provider, women are stuck in oscillation between either continuing education and having a career or adopting traditional gender roles of being a wife and mother. Not measuring up to gendered norms and expectations translates to other gendered stereotypes, such as being a “loser,” a “loafer,” “vagrant” and thus, a danger to society for men; and for women, to being a “spinster,” a “maid of all work,” unable to “keep a man” and thus, a burden to society. NEETs, especially those from a lower-income group, are bound to live with their families. With the ever-presence of male authority figures (i.e., father and brothers), under the surveillance and control of the family, NEETs’ experiences vary immensely. NEET women are mostly expected to be at home; thus, this expectation limits their socialization to their extended family and neighbors. Contrarily, men are expected to be outside of home during daytime; hence, NEET men are forced to spend their time on the street, as pack of men—something similar to adolescent male homosocial practices (bantering, loafing around, etc.), and unable to meet the ideal of socially respectable adult men. This results in anger, frustration, and shame (Maguire, 2021), and feeling under the scrutiny of both their families and society in general. They also become stigmatized as “vagrant” men and a security issue by the larger society. S3 illustrated how dramatically societal issues can cascade due to the prevalence of gender stereotypes and expectations and how these constitute barriers against not just women’s but also men’s capabilities. For queer men, these barriers and resultant isolation are experienced even more dramatically as socializing within such male homosocial groups is not possible or preferable considering the rigid gender policing, bullying and homophobic violence. This led the S3 team to expand their scope to accommodate this variance in real-life experiences.

The generative tools for S4 were carefully developed not to inherently reproduce gender bias, however, they highlight the complexity of the roles and impact of gender stereotypes, expressions and identity for different subject positions. Their design research revealed various patterns and in-depth reflections relevant to relationships between parents and among family members, gender roles imposed within the family, gendered stereotypes of occupations, and gendered designations to/orientations toward objects, which all generate unique subject positions experiencing all aspects of life differently. Yet, the way individuals perceive satisfaction from life is influenced by these factors, such as being (un)happy or (un)successful, and not always explicitly but mostly implicitly filtered through gender stereotypes. For example, the pilot of *HowUFail* with 14 participants revealed that an unsuccessful, young woman is mostly assumed unmarried or divorced and without children, whereas an unsuccessful, young man is assumed unemployed, without friends and suffering from unrequited love.

All projects involved the recognition of diverse, intersectional subject positions beyond homogeneous gender categories of women and men, experiencing the identified societal issues differently. Accordingly, gender interests also vary greatly for these subject positions in different contexts. However, throughout the design processes, untangling gender interests from gendered norms and expectations remained a persistent challenge. This was due to two reasons, as we observed. Firstly, while they were trying to address gender interests, the beneficiaries of the design solutions had investment in existing gender norms, roles and expectations and the students were faced with the challenge of decoupling these expectations from gender interests as these tend to be highly imbued and intertwined. For example, while the women participating in the sales events (S2) enjoyed the monetary gains and the sense of competitiveness and achievement in the market through these activities, they maintained the perception of housewife duties and motherhood as virtues of women. This rigid orientation toward such gendered norms resulted in uneasiness for designers in this case, as the women’s communities they aimed to support the empowerment of did not seem to be that interested in economic independence. Secondly, straddling between their practical interventions and their critical stance resulted in implicitly and heuristically returning to gendered norms and expectations in certain aspects. This indicated that the students were oriented toward offering an immediately applicable remedy to certain identified problems. For example, a preliminary idea for S1 was an automat where women could purchase scarves and t-shirts while using public transportation to cover their clothes and avoid unwanted attention. Apparently, this would just reinforce existing sexism; and the students discarded this idea through a thorough reflection on the ways it would entrench norms and stereotypes during class sessions. However, such ideas are opportunities, rather than a handicap, in such pedagogical processes to encourage in-depth reflection on the impact of design outcomes on gender issues. In such cases, it becomes difficult to distinguish between needs arising from existing gender roles and expectations and long-term gender interests toward achieving GE and to address varying gender issues, especially when conducting design research to understand people’s needs and preferences or designing to respond to them. This indicates that disorientation during the design process does not directly translate to reorientations toward novel, innovative, and empowering outcomes, especially when negative capability is not developed. On the contrary, it might be met with backlash and reinforce readily existing orientations of both designers and beneficiaries of their designs.

The perseverance of existing gender norms and expectations may lead to frustration or pessimism for designers who set out to achieve GE in terms of strategic gender interests. This reminds the authors of the tension between second-wave feminism and the “housewife myth,” in which the former perceives the latter as the main obstacle against women’s self-actualization and argues for its denouncement. However, this does not necessarily need to be experienced as tension that cannot be resolved as the designers felt, and rather points toward co-existing but conflictual and competing practices, discourses, thoughts, feelings and desires that provide creative space for women’s self-actualization and harbor the potential for reorientation (Johnson and Lloyd, 2004). Here emerges the need to recognize empowerment as inclusive of both formal and informal processes, and both practical and strategic interests within the existing sociocultural setting, and that such cultural transformation is long-term and never straightforward. It sometimes requires taking detours and risking getting lost, but potentially leads toward innovative reconciliation. Directly challenging gender norms and roles might be considered threatening and undermining the gains of women’s social positions, relationally embodied through informal social networks and everyday life practices; however, these norms and roles might also be regarded as stepping stones for further cultural challenges. It is important to keep in mind that this process is ongoing back-and-forth reconciliation in different situations as reorientation, and opportunities and barriers should be considered with these in mind.

### 5.2 Addressing the intersectional structure of power and inequality

Gendered norms, expectations and stereotypes are shaped and gain their meanings within intersectional power relations, and their impact on individuals varies greatly according to their intersectional subject positions. Hence, questioning gendered norms and stereotypes should involve the recognition of their impact on intersectional subjectivities. During the process, intersectionality was deployed at design research and ideation stages with varying degrees and impact. For example, for S1, while the students recognized that women are using public transportation more than men, they also realized that its users were also either young (still in education, early career, or unemployed) or elderly and from lower income groups. Furthermore, they revealed a gendered sense of security in mass transportation, reflecting different priorities and strategies deployed by women and men. Women tended to sacrifice time and wait for less crowded busses to feel safer, and bring extra clothing (e.g., t-shirts and scarves) with them on the bus if they are dressed up for any reason, just to wear it on top of their outfits and try not to attract unwanted attention and sexual advances. Men, on the other hand, disclosed a sense of restlessness and heightened concerns for either being misjudged in any kind of social interaction (even in eye contact) as potential perpetrators (Harush et al., 2023) or witnessing an act of harassment and feeling obliged to perform chivalry by intervening since men are expected to protect others. This revealed the need to consider *intersectional situations* with critical perspectives, beyond fixed identity categories and subject positions, and in terms of dynamic and interactive spaces and their gendered aspects and meanings, such as the meaning of personal safety in public transportation beyond limited and abstract frames of interactional situations. This can provide a sociological and political imagination connecting personal troubles to wider societal issues that underline varying social harms beyond individualistic and isolated concerns of personal safety. Connecting the social harm perspective with a gendered sense of security requires how gender scripts and gender hierarchy affect the perception and experience of security and insecurity for different groups of people. Here, social harms involve social distance, mistrust, varying gendered prejudices and stereotypes, which entrench discrimination and alienation during social encounters, and help us contemplate the meanings of feeling insecure and anxious rooted in the hetero-patriarchal society. In relation to this, S1 also reveals the meaning of absence in data on *trans* and gender non-conforming people, who do not use public transportation to avoid potential harassment, violence, and discrimination.

For S2, the students recognized that the women’s community they targeted mostly consisted of women outside the paid labor force, under-educated, from lower income groups and relatively newer residents of the city living in the periphery, whereas highly educated, white-collar, middle-class women were not involved in their activities. This demonstrated that women are not a homogenized category, and their experiences differ depending on class, education, and age. Furthermore, the community members regard the municipality’s sales events as income sources and focus more on the monetary gains, self-promotion, and competition among them rather than on solidarity and collective empowerment. Therefore, the idea of individualistic empowerment within a neoliberal market economy oriented around existing gendered norms creates a fundamental barrier against the empowerment of the whole community and results in the marginalization and exclusion of women who are elderly, illiterate, in extreme poverty, from ethnic minorities, or *trans*. All the design interventions for S2 were developed to overcome this and achieve both individual-level and community-based empowerment more inclusively, by addressing this separation, polarization and social distance among women signifying indifference toward each other’s problems, and lack of interaction, cooperation, and solidarity in resolving them.

For S3, the students critically untangled NEET as an inherently marginalizing category encompassing multiple intersectional identities based on age, level of education, socioeconomic status, dis/abilities, gender and sexual orientation, thus effectively representing interlocking domains of oppression. In addition, these intersectional identities of NEETs are widely excluded from social policies. Furthermore, this is experienced dramatically differently by different subject positions and indicates gendered meanings and impacts of being NEET. The intersectionality approach was especially useful in identifying various domains of oppression, not only for women but also for men. The concepts of subordinate and marginalized masculinities were utilized in alignment with the intersectionality approach to make sense of NEET men’s experiences. Not measuring up to the ideal of socially respectable adult men, NEET men’s loitering on the streets is coded as anti-social behavior, attracting the attention of state agents as suspicious, potentially criminal individuals; thus, falling under enhanced surveillance by the police, such as frequent “random” ID checks. On the other hand, as unmarried, not working and young, NEET women’s spending time in the public sphere is frowned upon and codified as indecent within the frame of gendered moral and honor codes in culture. This restricts their mobility, autonomy and ability to accumulate social capital beyond their immediate social environment (e.g., family, neighbors), further aggravating the injustice and inequality they experience. In the face of such diverse forms of social isolation, the S3 team developed alternative digital and physical spaces to reach out to and accommodate NEETs, with different design strategies. Different from other projects, in S4, the students used the intersectionality approach as an inquiry technique (Hill Collins and Bilge, 2020) as part of their generative tools. As we explained in the previous section, their purpose was to reveal the intersectionally changing meanings of success and failure, and satisfaction in life for different fictional subject positions.

Even if minorities, migrant/refugee groups, and LGBTQ+ groups were all theoretically and conceptually acknowledged in all projects, the students found it challenging to address these groups adequately in their research and design practices. It would be unfair to explain this only through implicit bias or a shortcoming of students’ capabilities to deploy a critical and intersectional gender lens rather it indicates the hardness of reaching out to such groups that are already disoriented within the existing system, collecting data about and for them, properly analyzing their problems, and developing solutions for them (Guyan, 2022). This shows the importance of not only a critical, inclusive, and intersectional gender lens beyond the gender binary but also finding ways to reach out to what is missing in the data instead of accepting it as a limitation. In this sense, recognizing what is missing through an intersectional approach may instigate designers to reorient toward them.

### 5.3 Addressing empowerment beyond the market-oriented, individualistic neoliberal order

Although the intersectional structure of power and inequality were critically analyzed, the challenges arising from the hegemony of the neoliberal order inadvertently affected design education and practice when students were developing design interventions. The fine line between the empowerment of individuals and the empowerment of communities proved harder to navigate for all projects. For example, for S1, the students captured robust data about the bus transport experience revealing the gendered aspects of the fear of crime, which could have led to the problematization of this issue to pursue a gendered sense of security in relation to long-term strategic gender interests of varying subject positions. Instead, they proposed an absolute fix with a design-against-crime approach to alleviating the individual sense of insecurity and soothing people’s anxieties about mobility by ignoring its dynamically intersubjective nature. They did not really reflect on what security means, and for whom, and focused more on preventing what is considered a “crime” while disregarding the social harms around public transportation they revealed during data collection, such as mistrust between strangers in public, social distance and discrimination based on prejudice and stereotyping especially toward, e.g., migrants and refugees, and disempowering aspects of gender stereotypes, expressions and expectations during mobility that limits women and men’s behaviors and attitudes differently in relation to the gendered sense of security (Fenster, 2005). Ultimately, their intervention was an additional layer of security measures, addressing the fear of crime as-is without questioning social harms or a gendered sense of security, let alone the absence of gender non-conforming people during their design research.

The process for S2 started out as developing interventions for community-based empowerment, theoretically rooted in Escobar (2018) autonomous communities, and attempted to build solidarity among community members and transform it as more inclusive of other women. Design interventions were developed accordingly to raise awareness of the community about commonalities in their everyday lives in terms of unpaid, emotional labor, to build a culture of collaboration and solidarity through the co-production of artifacts, and to initiate an online community for knowledge sharing and collaboration. However, the students developed a series of tools and services for skills building not only in crafts but also in business development, online marketing and promotion, all of which were more focused on the neoliberal individualistic empowerment narrative and missed the opportunity to deploy existing capacities of women in creativity, innovation and management that remain invisible and mostly undervalued, regarding domestic work as repetitive, mundane and unproductive in comparison to capitalist, market-oriented labor force valued in public and regarded as productive (Johnson, 2012). In an attempt to facilitate a more collaborative and inclusive community that can overcome structural barriers against women’s empowerment, such comparison remained tacitly embedded in design thinking and solutions due to the profession’s foci on industrial production and marketing, and it turned out to be an obstacle for designers when it came to recognize, re-value and make visible the existing capacities of these women including innovation and management skills in order to reveal their potentials for empowerment.

For S3, the students initially adopted gender mainstreaming with an apolitical approach, but then they discovered the political aspects surrounding NEETs—especially that the term is framed around a *developmental* perspective on human rights and a neoliberal sense of individual, economic empowerment, which disregards people’s hopes, wants, needs and capabilities. In turn, they began to analyze this issue as another form of *slow violence* of the neoliberal order (Nixon, 2013) that equates success to economic power, entrenches gender stereotypes and results in immense oppression that temporally stretches and encompasses NEETs’ lives from childhood. This, coupled with the affective rhetoric based on a neoliberal and gendered emphasis on passion and perseverance (Harvey and Shepherd, 2016) in addition to disquieting material conditions, creates immense affective barriers against NEETs’ empowerment and dramatically exacerbates their circumstances. In a noteworthy attempt during idea generation, they deployed the *capabilities* approach to human rights (Nussbaum, 2011) suggesting that people are not merely entitled to some rights but they should be capable of flourishing. Nevertheless, this approach also revealed certain in-capabilities for both designers themselves and NEETs, experienced as disorientation (Ahmed, 2006), i.e., discomfort, helplessness, feeling lost and failure. The designers tried to find ways to both endure the disorientation by developing a negative capability and navigate out of it by reorienting themselves. They embraced their limited roles as mediators and facilitators that both recognized their own disorientation in the face of the problem as designers in the field and recognized NEETs’ disorientations and their feelings of discomfort about being disoriented in a larger society. Accordingly, they set out to develop design outcomes that can humbly provide NEETs with different paths for enduring such disorientation and exploring ways out of it. Having said that, when further detailing their ideas, they felt the need to utilize personal stories, role models and similar narratives of neoliberal individualistic empowerment, which are especially inaccessible to most queer people, rather than further exploring the fertile ground for NEETs developing self-reflexivity and negative capability that might have been enabled through such disorientation. This was one of the most striking tensions observed in this project.

## 6 Discussion and conclusion

The theory-informed design process and resultant design outcomes allowed the authors to explore potentials for incorporating gender lenses throughout the design process, revealing various opportunities as well as practical implications for gender-sensitive design. The students principally adopted gender theories and concepts—such as gender needs and interests, intersectionality, gender as a more inclusive term, gender as an analysis category to reveal unequal power relations and social injustices—to develop norm-critical perspectives. Especially, how they critically reflected on their own engagement and developed design outcomes not to intervene from the outside but rather to facilitate empowerment from within was prominent for all the students. This involved a critical reflection on their existing design practices and a shift in perception of their roles as designers from creator/problem-solver to participant/facilitator. A similar shift was observed for the design outcomes as well. Throughout the process, they critically assessed the opportunities and limitations of any design outcome they might develop. This led to the development of design outcomes as *intermediaries* that would facilitate *innovative reconciliations* and users’ empowerment from within to tackle gender inequality and injustice, rather than “total” solutions attempting to solve multifaceted aspects of such complex, interrelated societal/cultural issues and social harms. Such *intermediaries* can incorporate sociological and political imaginations and potentially respond to *intersectional situations* that are inherently dynamic, interactive, and intersubjective.

The biggest resistance observed was straddling their existing orientations, emerging disorientations during the process, and finding novel paths of reorientation, especially when the students were transferring their design research into practical design outcomes. When emerging disorientations cannot be contained with negative capability (which can enable novel directions of reorientation), they can instead result in reorienting back and even reinforcing already existing orientations. Gaps and problems that emerged throughout their research steered the students to hastily generate solutions; however, the problems identified were not grounded as much in some cases and risked the under-utilization of their design research for a more effective design process. This tension between the need for in-depth, critical reflection in all steps of the process and the tendency to hastily generate and ascribe solutions sometimes resulted in missing out on such reflection. Practical gender interests which might be intertwined and mostly confused with culturally accepted and embodied gendered norms, roles, expectations and affections sometimes overshadowed strategic gender interests (e.g., GE, justice, and empowerment). This reveals a dilemma about the design process that aims to recognize and address gender issues, and points to a need for embedding gender as an analysis category, with all its critical potentiality, in the overall design curriculum, especially in courses related to managing the design process.

The conflict between market-driven design education and the need for more inclusive, participatory and unending design processes to respond to such societal and cultural issues resulted in ambivalence for students, as the tension between the theoretical/ideal approaches and practical/realisable outcomes resulted in frustration and disappointment at times. Additionally, the socio-political landscape of Turkey with its increasingly oppressive gender regime and the rise of anti-gender politics undermining women’s and LGBTQ+ rights on the one hand, and the ongoing local struggles of civil society organizations, advocacy groups and local communities to empower women and LGBTQ+ people on the other, contributed to this frustration further. We observed the tendency that this might actually lead to disempowerment for designers in the form of disorientation, as they may not be able to identify how their skills and capacities can be useful in the face of such complex issues, and lead toward a purely theoretical, critical stance not translating to their design processes and outcomes—and the “critique can become merely an expression of profound cynicism, which then works to sustain dominator culture” (hooks, 2003, p. 15). However, we also observed that such ambivalences and disorientation pose opportunities for future designers to re-orient their design processes and find novel ways of engaging with social and cultural issues locally, especially through reflexivity and negative capability that they can develop.

The process involved the students candidly reflecting on, negotiating and dismantling the gender stereotypes they implicitly embody and deploy, which was inevitably time-consuming and required considerable emotional work. Yet this is also a promising aspect of this educational design project in terms of learning outcomes, as they have acknowledged the importance of a continuous, never-ending process of self-reflexivity (Migdalek, 2020) in their future design practice. This further emphasises the need for such educational design projects during which future designers can more freely explore and develop norm-critical gender lenses in their design processes. While we presented an initial attempt at this, what skills and capacities from more traditional and mainstream design professions are transferable and applicable, and what needs to be dismantled, unlearned and reconfigured remain open questions.

Finally, we cannot gloss over the fact that both students and instructors are gendered subjects, and, thus, we should acknowledge our gendered needs, interests, expectations, emotions, investments, and practices as well. The self-reflexivity sessions allowed in-depth reflection on these, including biographical elements and personal experiences within varied contexts along with the projects. Although instructors and students are positioned in hierarchical positions within the higher education structure, we strived to create a safe space for such conversations to occur. We believe creating such safe spaces is essential not only for a gender-sensitive approach to design education and practice but also for initiating a cultural transformation.

## Data availability statement

The original contributions presented in the study are included in the article/Supplementary material, further inquiries can be directed to the corresponding authors.

## Ethics statement

Ethical approval was not required for the studies involving humans because the design research procedures of the students as part of the course were evaluated and approved for ethical compliance by the instructors, as per the institutional guidelines. The studies were conducted in accordance with the local legislation and institutional requirements.

## Author contributions

EY: Writing – original draft, Writing – review & editing. YB: Writing – original draft, Writing – review & editing.

## Footnotes

^1^We should note that the official name of the country has been changed to “Türkiye” via the United Nations in 2021. However, we decided to use “Turkey” since the name of the country is still commonly known as such.

^2^Negative capability is a term coined by Keats (2011) and refers to the ability to tolerate uncertainty, particularly the active uncertainty that is to do with being without a template and yet being able to tolerate, or even relish, a sense of feeling lost. The authors propose that negative capability is fundamental to develop a creative and imaginative response to disorientation and provides a way of embracing uncertainty and openness for reorientation rather than seeking closure and absolute resolution.

^3^We believe it is important to keep in mind that anti-gender “movement” and anti-gender “politics” are closely related but they are not identical. The former is more about the actors and actions that they take, while the latter is more about ideologies and strategies. The latter is more relevant in the context of Turkey in terms of ideologies and strategies, however, their impact on and uptake by various actors within the society are not unequivocal.

^4^The project syllabus is provided as Supplementary material, which also lists all the readings assigned to students.

^5^The educational outcomes of this study should not be confused with the students’ design outcomes. This paper focuses on the educational outcomes of this case. While students’ design outcomes are important for the evaluation of this educational case, they remain secondary in comparison to the educational outcomes this manuscript focuses on. In this sense, we are only presenting and utilising the student design work to contextualise, analyse and assess the educational case.

^6^Halberstam’s (2011) queer art of failure is a concept that challenges the conventional notions of success and failure in a heteronormative, capitalist society. He argues that failure can be a productive way of critiquing and resisting the dominant systems of power and knowledge and explores how failure can open up more creative, cooperative, and queer ways of being in the world. This is not about giving up or being pessimistic, but rather about finding alternatives to the normative definitions of success that are based on individualism, conformity, and profit.
