# Supplementary material for: Dis/re-orienting design through norm-critical gender lenses: an educational case in Turkey
Source: Front Sociol. 2024 Mar 28;9:1341091. doi: 10.3389/fsoc.2024.1341091 (PMC11007197; doi:10.3389/fsoc.2024.1341091)
Supplement: Supplementary file 1 [file Data_Sheet_1.PDF]

Supplementary Material for 'Dis/Re-orienting design through norm-critical gender lenses: An educational case in Turkey'

The below course/project syllabus was provided to the students at the beginning of the educational case presented in this manuscript.

MIDDLE EAST TECHNICAL UNIVERSITY
DEPARTMENT OF INDUSTRIAL DESIGN

Çankaya
MUNICIPALITY

ID501 Advanced Project Development in Industrial Design

2020-21 Spring / Tuesdays, 13:30-17:40 & Thursdays, 8:40-13:30 / Online

Assist. Prof. Dr. Yekta Bakırlıoğlu, Instructor Dr. Erman Örsan Yetiş

Theme: Gender-sensitive Design

This course aims to build designer-ly skills beyond the conventional design process and equip you with the capability to negotiate your own roles as *agents of change*, who can envision futures with an inclusive and collaborative perspective. This is a multifaceted, tough yet rewarding challenge, as it involves a thorough understanding of the current situation, comprehension of possible future directions and recognition of opportunities and setbacks along the way.

This year, our theme for ID501 is 'gender-sensitive design'.

In the context of this course, 'gender-sensitive design' refers, firstly, to integrating gender equality endeavours into the design process and solutions in line with gender mainstreaming from a macro perspective. It envisions a design process where gendered practices, social issues and inequalities are thoroughly recognized and effectively addressed by designers and design researchers. Secondly, it refers to critically adopting some contemporary gender debates, theories and methodologies in order to build a comprehensive critique of, and to transform accordingly, design practice and theory.

Gender equality has become more and more discussed as an issue of utmost importance both in academic literature and through programmes initiated by inter-governmental organizations, such as UNWOMEN and EIGE. It is also Sustainable Development Goal #5 and aims to lead sustainable cultural transformation to end all forms of gender-based discrimination and to empower women and girls.

Social sciences and humanities literature has been tackling the issue of gender inequality, espousing various modes of resistance, revealing unequal practices in society and advocating ways of tackling the issue on multiple fronts. Especially prominent in the EU legislation, gender mainstreaming and the intersectionality approach are the state-of-the-art strategies providing researchers, policymakers, activists, etc. with the necessary tools and methods to tackle gender inequality and to build robust,

concrete responses. Similarly, the EU's research and innovation support programmes now require concrete evidence of how the 'gender aspect' is interwoven into project proposals.

All these indicate that we, as designers, need to critically reflect on design research and practice and adopt a gender-sensitive perspective as an irreplaceable aspect of design. We will do as such on two levels:

1. **By rethinking every stage, tool and method for our design process.** We will familiarize ourselves with contemporary concepts in gender theory and approaches to tackling gender inequality, critically reflect on the design process, and adapt our process towards 'gender-sensitive design'.

2. **By tackling problem areas where gender inequality persists.** In collaboration with Çankaya Municipality, Women and Family Services Unit, we will scope our design project among many problem areas present in our city and develop design solutions addressing those issues. This endeavour requires us to look beyond product/service design and question our role as designers, as well as the roles of our designs on a meta-level, in relation to existing stakeholders and mechanisms in society.

Objectives and Learning Outcomes

ID501 aims to foster:

- Understanding of the current, emergent and future directions of production and consumption, as well as the emerging roles and competencies of designers, their collaborators, and users in the design process,
- Recognition of the steps to be taken to reach a 'desired' future through a cross-disciplinary perspective,
- Skills and experience in planning design and research activities that target specific issues and contexts of use,
- In-depth understanding of and critical reflection on possible designer-ly interventions for transitioning towards desired futures from a social and political perspective,

On the completion of ID501, students will be able to:

- Build robust visions through the analysis of the state-of-the-art in various disciplines,
- Identify their roles in the future of the profession and recognise their capacity as *agents of change*.
- Identify the paths to 'desired' societal and technological changes,
- Transform findings from literature to design explorations through a critical socio-political perspective,
- Critically reflect on (un)intentional socio-political aspects of the design process and outcomes.

Structure of the course

Stage 1: Building a 'gender-sensitive design' approach

At this stage, we will read *a lot* and the whole class will work as a team. The purpose of this stage is three-fold:

1. Understanding 'gender' through a search and review of literature from various disciplines, and gray (non-academic) literature,
2. Understanding the changing roles and capabilities of designers and design process,
3. Building a critical socio-political perspective through contemporary social theory.

At the end of this stage, you are expected to:

- Visualise your critical approach through a **mind map**,
- Narrate the vision you envision with reference to literature, giving reference to various forms of activity, various actors and their roles in this future (e.g. professions, communities, companies, institutions, infrastructure, etc.)
- Identify your own role considering your existing skills and capabilities.

Stage 2: Scoping 'gender inequality' in Çankaya

You will work as design teams at this stage, in which you will deploy the critical socio-political perspective you built to carry out field research. This can involve design ethnography, expert interviews, user research and/or surveys. We will decide on the methods to deploy as a class and assign smaller teams to carry out various research tasks.

At the end of this stage, you are expected to:

- Identify the specific problem area(s) we will focus on as a class
- Develop more specific **design briefs** as separate teams – the briefs of separate teams should be complimentary with each other, addressing the overall problem area.

At this stage, we will also need to decide on a way to present this in a more engaging manner.

Stage 3. Design your intervention

You will develop design interventions individually responding to your team's design brief. Each one of you will envision and design a product and/or service for an intervention point of your team's design brief. You will need to ground your design decisions with the literature and present its relationship to others' designs in your team.

This is the tricky part! *You are expected to design something conceptually contributing to your team's design brief, but also consider how these designs contribute to each other.*

Stage 4: Exhibition

We will showcase our work (might be a digital showcase or a physical exhibition, depends on pandemic measures)

Schedule

March 16 - Introduction
Handbooks to keep throughout the course:
Council of Europe. (2016). *Gender Equality Glossary*.
Kothari, A., Salleh, A., Escobar, A., Demaria, F., & Acosta, A. (Eds.). (2019). *Pluriverse: A Post-Development Dictionary*. Retrieved from http://cup.columbia.edu/book/pluriverse/9788193732984

March 18 - Presentations on **different standpoints on gender equality & gender mainstreaming**
Crenshaw, K. (1990). Mapping the margins: Intersectionality, identity politics, and violence against women of color. Stan. L. Rev., 43, 1241.
In: McCann, C. & Kim, S.K. [Eds.] (2017). Feminist Theory Reader (Fourth Edition). London: Routledge
- Nash, J. C. Re-Thinking Intersectionality, pp. 194-203
- Patil, V. From Patriarchy to Intersectionality: A Transnational Feminist Assessment of How Far We've Really Come, pp. 204-212

March 23 - Presentations on **sustainability and environmental crisis through a gender lens**
Cornwall, A.& Rivas, A. M. (2015) From 'gender equality and women's empowerment' to global justice: reclaiming a transformative agenda for gender and development, *Third World Quarterly, 36*:2, 396-415, DOI: 10.1080/01436597.2015.1013341
Plumwood, V. (1993). Feminism and Ecofeminism. *Feminism and the Mastery of Nature.* London: Routledge. pp. 19 - 40.
Requena-Pelegri, T. (2017). Green Intersections: Caring Masculinities and the Environmental Crisis. In: Armengol, J. M., Bosch-Vilarrubias, M., Carabi, A. and Requena-Pelegri, T. [Eds.] Masculinities and Literary Studies: Intersections and New Directions, pp. 143-152
Hultman, M. & Pule, P. (2020) Ecological Masculinities: a response to the Manthropocene question?. In: Gottzen, L. Mellström, U, Shefer, T. [Eds.]. *Routledge International Handbook of Masculinity Studies.* London: Routledge, pp. 477 - 487.

March 25 - Presentations on **bodies and objects within a gender perspective**
Haraway D. (1991) A Cyborg Manifesto: Science, Technology, and Socialist-Feminism in the Late 20th Century. In *Simians, Cyborgs and Women: The Reinvention of Nature.* New York; Routledge, pp.149-181
Puar, J. K. (2012). "I would rather be a cyborg than a goddess": Becoming-intersectional in assemblage theory. PhiloSOPHIA, 2(1), 49-66.
In: Ahmed, S. (2006). Queer Phenomenology: Orientations, Objects, Others. Duke University Press
- Introduction: Find Your Way, pp.1-24
- Orientations Toward Objects, pp. 25-64

March 30 - Presentations on **bodies and objects within a gender perspective**
Gottzén, L. (2011). Metaphors of masculinity: Hierarchies and assemblages. GEXcel work in progress report, 15, 229-239**.**
Balkmar, Dag and Mellström, Ulf "Masculinity and Autonomous Vehicles: A Degendered or Resegregated Future System of Automobility? *Transfers: Interdisciplinary Journal of Mobility Studies 8*(1), Spring 2018, Special Section on Degendering the Driver.
Young, I.M. (1980). Throwing like a girl: A phenomenology of feminine body comportment motility and spatiality. Hum Stud 3, 137–156. https://doi.org/10.1007/BF02331805
Braidotti, R. (2003). Becoming Woman: Or Sexual Difference Revisited. Theory, Culture & Society, 20(3), 43–64. https://doi.org/10.1177/02632764030203004

April 1 - Presentations on **bodies and objects within a gender perspective**
In: Behar, K. [Ed] (2016). Object-oriented feminism. Mineapolis: University of Minnesota Press
- Katherine Behar, An Introduction to OOF,
- Irina Aristarkhova, A Feminist Object,
- Timothy Morton, All Objects Are Deviant: Feminism and Ecological Intimacy,

Berlant, L. (2006). Cruel Optimism. *Differences, 17*(3), 20–36. https://doi.org/10.1215/10407391-2006-009
Baraitser, L. (2015). Touching Time: Maintenance, Endurance, Care. In: Frosh, S. [Ed.] Psychosocial Imaginaries: Perspectives on Temporality, Subjectivities and Activism. New York: Palgrave Macmillan. 21-47.

April 6 - *Çankaya Municipality, Women and Family Services Unit Presentation [in Turkish]*
Presentations on **design, gender, social innovation, pluriverse**
Kaygan, P. (2016). Gender, Technology, and the Designer's Work: A Feminist Review. *Design and Culture, 8*(2), 235–252. https://doi.org/10.1080/17547075.2016.1172862
Forlano, L. (2017). Posthumanism and Design. *She Ji, 3*(1), 16–29. https://doi.org/10.1016/j.sheji.2017.08.001
A chapter of choice in: Manzini, E. (2015). Design, When Everybody Designs: An Introduction to Design for Social Innovation. MIT Press

---

April 8 - Presentations on **design, gender, social innovation, pluriverse**
In: Mareis, C., & Paim, N. (Eds.). (2021). Design Struggles: Intersecting Histories, Pedagogies, and Perspectives. Amsterdam: Valiz.
- Autonomous Design and The Emergent Transnational Critical Design Studies Field, Arturo Escobar, 25–38
- Made in Patriarchy II: Researching (or Re-Searching) Women and Design, Cheryl Buckley, 43–57
- Not a Toolkit: A Conversation on the Discomfort of Feminist Design Pedagogy, Griselda Flesler *in conversation with* Anja Neidhardt and Maya Ober, 205–225
- Design Justice: Towards an Intersectional Feminist Framework for Design Theory and Practice, Sasha Costanza-Chock, 333–353e.

April 13 - Presentations on **methodological concerns on gender-sensitive research**
Readings TBA. Topics to be tackled: Feminist & gender-sensitive research, Self-reflexivity & positioning, Gender-sensitive ethnography, Intersectionality & plurality, Data analysis

April 15 - Gender-sensitive design approach mind-map

April 15 - May 13 - **Field Research**

May 13 - Pre-jury
- **Gender-sensitive design approach mind-map**
- **Field Research Outcomes**
- **Design briefs**

May 13 - June 10 - **Design your intervention**

June 10 - June 24 - **Exhibition/Showcase Design**
**June 24 - Jury**
